# Repeatability of Spectral Domain Optical Coherence Tomography Measurements of Bruch’s Membrane Opening-Minimum Rim Width in Epiretinal Membrane Patients with Peripapillary Involvement

**DOI:** 10.3390/jcm10112240

**Published:** 2021-05-21

**Authors:** Ki Yup Nam, Bum Jun Kim, Woo Hyuk Lee, Yong Seop Han

**Affiliations:** 1 Department of Ophthalmology, Chungnam National University Sejong Hospital, Sejong 30099, Korea; oksnam1231@daum.net; 2 Department of Ophthalmology, College of Medicine, Chungnam National University, Daejeon 35015, Korea; 3 Department of Ophthalmology, Gyeongsang National University Changwon Hospital, Changwon 51472, Korea; deankim21c@gmail.com (B.J.K.); lwhyuk@naver.com (W.H.L.); 4 Department of Ophthalmology, College of Medicine, Gyeongsang National University, Jinju 52828, Korea; 5 Gyeongsang Institute of Health Sciences, Gyeongsang National University, Jinju 52828, Korea

**Keywords:** Bruch’s membrane opening-minimum rim width, retinal nerve fiber layer, repeatability, epiretinal membrane

## Abstract

The Bruch’s membrane opening-minimum rim width (BMO-MRW) is a recently introduced parameter of the neuroretinal rim. We analyzed the repeatability of spectral-domain optical coherence tomography (SD-OCT) measurements of BMO-MRW in epiretinal membrane (ERM) patients with peripapillary involvement, since the surface around the optic disc is distorted in such patients. BMO-MRW and retinal nerve fiber layer (RNFL) thickness measurements were performed using SD-OCT in prospectively enrolled ERM patients and age-matched healthy control individuals. After two consecutive measurements with a 5 min interval, repeatability was analyzed using the intraclass correlation coefficient (ICC), repeatability coefficient (RC), and coefficient of variation (CV). Fifty-two eyes of 52 ERM patients and 62 eyes of 62 healthy controls were included in the study. The ICCs of the mean BMO-MRW/RNFL thickness measurements were 0.999/0.985 in ERM eyes and 0.999/0.999 in normal eyes, respectively. The RC values of mean BMO-MRW/RNFL thickness measurements were 9.0/6.25 μm in ERM eyes and 4.61/0.92 μm in normal eyes, respectively. The CV values were 0.91% and 1.45% for BMO-MRW and RNFL thickness in ERM eyes, and 0.63% and 0.33% in normal eyes, respectively. In ERM eyes, the RC, CV of average BMO-MRW were 1.9 and 1.4 times greater than those of normal eyes, but 6.8 and 4.4 times greater for average RNFL thickness. BMO-MRW and RNFL thickness showed good repeatability in the diseased eyes with peripapillary involvement and healthy control eyes. Based on the ICC, RC, and CV values, the repeatability of BMO-MRW measurements in peripapillary membrane patients was better than that of RNFL thickness.

## 1. Introduction

Bruch’s membrane opening-minimum rim width (BMO-MRW) is a recently introduced measurement parameter of the neuroretinal rim. Reis et al. [1] defined BMO as the optic nerve head rim boundary and introduced the minimum distance from the BMO to the internal limiting membrane (ILM) as a new factor for optic disc analysis. Several studies reported that BMO-MRW has higher diagnostic utility for glaucoma than the rim area measured by confocal laser tomography or retinal nerve fiber layer (RNFL) thickness measured by optical coherence tomography (OCT), and it has a better correlation with the results of visual field examination [2,3,4,5,6].

Spectral domain optical coherence tomography (SD-OCT) parameters, such as BMO-MRW and peripapillary RNFL thickness, may show discrepancies in the same patient, and these parameters may also show differences among repeated examinations in the same patient.

Epiretinal membrane (ERM) is an age-related retinal disease, and the incidence of glaucoma also increases with age. Primary angle-closure glaucoma has a prevalence of approximately 5% in patients ≥ 40 years old, and ERM is present in 2–10% of these patients [7,8,9,10,11,12]. If ERM involves the peripapillary area, automatic segmentation of the ILM may be affected by changes in the retinal surface.

Repeatability is essential for a test to be clinically useful. The intraday and intravisit repeatability of BMO-MRW measurements were reported to be good in subjects both with and without glaucoma [13,14]. However, the subjects in these studies had no retinal diseases, which is important because these can alter the retinal surface and potentially affect the BMO-MRW. Peripapillary ERM may affect the repeatability of BMO-MRW measurements.

We assumed that peripapillary ERM may affect the repeatability of BMO-MRW measurements; thus, this study was performed to analyze the repeatability of BMO-MRW measurements in patients with ERM with peripapillary involvement, which can cause changes in the retinal surface. We also compared the repeatability with RNFL thickness and normal patients.

## 2. Materials and Methods

This prospective study was approved by the Institutional Review Board of Gyeongsang National University Changwon Hospital, Gyeongsang National University School of Medicine (approval No. GNUCH 2018-08-011; approval date, 17 September 2018). The study adhered to the tenets of the Declaration of Helsinki.

Patients with idiopathic ERM involving the papillomacular region of the retina were enrolled. Written informed consent was obtained from all patients prior to enrolment and examination. Patients with other retinal diseases that can change the retinal surface, such as macular edema in association with diabetic retinopathy, retinal vascular occlusion or age-related macular degeneration, were excluded. Patients with other diseases and conditions that may affect BMO-MRW, such as glaucoma, myopia > −6.0 diopters, axial length > 26 mm, history of previous vitrectomy or uveitis, were also excluded. Healthy subjects who visited our clinic for an eye evaluation and had no ocular abnormalities were enrolled as controls. Data were obtained from one eye with images of higher quality score for each patient. We used G* Power software (version 3.1.9.6; Franz Faul, Universität Kiel, Kiel, Germany) to calculate the sample size [15]. G* Power calculations indicated that 51 subjects would be needed to compare the two consecutive measurements.

### 2.1. Optical Coherence Tomography Measurements

After inducing mydriasis by topical administration of 0.5% tropicamide and 2.5% phenylephrine, the disc area was measured on SD-OCT scans (Spectralis^®^; Heidelberg Engineering GmbH, Heidelberg, Germany) using Glaucoma Module Premium Edition software (Heidelberg Engineering GmbH). In total, 24 radial B-scans were obtained for the BMO-MRW measurements. A scan circle 3.5 mm in diameter among three scan circles was used for determination of peripapillary RNFL thickness. Well-centered scans with a quality score >20 were used. Data were collected on the individual-specific fovea–BMO (FoBMO) axis, defined as the axis between the center of the BMO and the fovea, which allows for more precise sectoral analysis of cyclotorsion compared to the conventional technique, and assessment based on a normative database.

**Figure 1:**
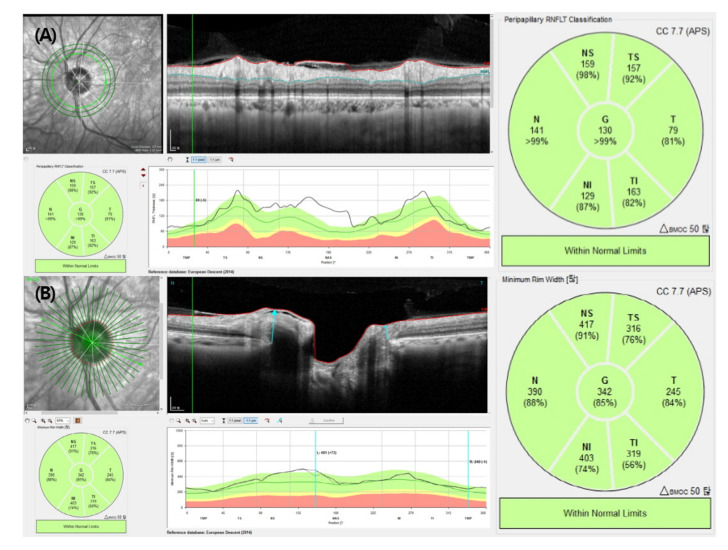
Retinal nerve fiber layer thickness (**A**) and Bruch’s membrane opening-minimum rim width (**B**) determined by spectral-domain optical coherence tomography. G: average; TS: superotemporal; T: temporal; TI: inferotemporal; NI: inferonasal; N: nasal; NS: superonasal.

We obtained average and sectoral (superotemporal, temporal, inferotemporal, inferonasal, nasal, superonasal) RNFL thickness (Figure 1a) and BMO-MRW measurements (Figure 1b). Repeatability was determined by comparing two consecutive RNFL thickness and BMO-MRW measurements in eyes with ERM, with a 5 min interval.

### 2.2. Statistics

Statistical analyses were performed using SPSS software (ver. 18.0; IBM Corp., Armonk, NY, USA). The paired t test was used for comparison of the two BMO-MRW and RNFL thickness measurements. Comparison of mean values between diseased and healthy control eyes was performed using the independent-samples t test for normally distributed data.

For the comparison of baseline characteristics between diseased and normal eyes, the quantitative and qualitative variables were analyzed by independent sample t test (normally distributed data) and Chi-square test, respectively.

The repeatability of the two consecutive BMO-MRW and RNFL thickness measurements was assessed using the intraclass correlation coefficient (ICC), repeatability coefficient (RC), and coefficient of variation (CV). The ICC represents the correlation between two variables measured at different time points (t); values range from 0 to 1, and higher values indicate better repeatability.

The RC can be calculated using the standard deviation (SD) of the difference between two measurements and is defined as:

1.96 × √2 × within-subject SD(1)

We expected the difference between the two measurements to be no greater than the RC value for 95% of subjects [16].

The CV, also known as the relative SD, is a standardized measure of dispersion of a probability distribution and is calculated as the within-subject SD divided by the mean, multiplied by 100 and expressed as a percentage [17]. A small CV value indicates that the variability of the difference between two consecutive measurements is also small, therefore indicating better repeatability.

## 3. Results

### 3.1. Baseline Characteristics

Fifty-two eyes of 52 patients with peripapillary membrane and 62 normal eyes of 62 control subjects were included in the study. There were no significant differences between the two groups in mean age (59.2 ± 13.5 and 58.6 ± 10.0 years, respectively, *p* = 0.843) or the male:female ratio (57.7%:42.3% in diseased eyes and 66.1%:33.9% in normal eyes, *p* = 0.355). The right eye was significantly more in diseased eyes (right:left ratio = 63.5%:36.5%) than normal (37.1%:62.9%, *p* = 0.005). The mean initial best-corrected visual acuity (BCVA, logMAR) and spherical equivalent were 0.15 ± 0.14 and −0.66 ± 2.09 diopters, respectively, in the diseased eyes and 0.28 ± 0.32 and −0.41 ± 1.66 diopters, respectively, in the normal eyes (*p* = 0.111 and *p* = 0.647, respectively). The mean central macular thickness (CMT) was significantly greater in diseased than normal eyes (333.4 ± 88.7 and 275.6 ± 45.2 μm, respectively, *p* < 0.001) (Table 1).

### 3.2. Average and Sectoral BMO-MRW and RNFL Thickness

The mean values of the two consecutive BMO-MRW and RNFL thickness measurements were compared between the diseased and normal eyes. The mean of average BMO-MRW obtained in the first and second measurements was 321.6 ± 107.0 and 320.8 ± 104.2 μm, respectively, in diseased eyes (*p* = 0.449), and 265.9 ± 49.4 and 266.0 ± 49.6 μm, respectively, in normal eyes (*p* = 0.770). In all sectors, the mean BMO-MRW measurements showed no significant differences in the diseased or normal eyes (Table 2).

The mean RNFL thickness of average values was 124.3 ± 42.5 μm and 124.8 ± 43.1 μm in diseased eyes, and 101.3 ± 13.6 μm and 101.4 ± 13.5 μm in the first and second measurements, respectively; the differences were not significant (*p* = 729 and *p* = 0.240, respectively). In addition, there were no significant differences between the two mean RNFL thickness values in any sector, in either group of eyes (Table 2).

### 3.3. Repeatability of Two Consecutive BMO-MRW and RNFL Thickness Measurements

The ICCs for BMO-MRW and RNFL thickness were compared between diseased and normal eyes. For eyes with a peripapillary membrane, the ICC for the average BMO-MRW was 0.999 (sectoral ICCs: 0.986–0.999). The ICC of the average RNFL thickness was 0.985 (sectoral ICCs: 0.986–0.995).

In the normal eyes, the ICCs for the average BMO-MRW and RNFL thickness were 0.999 and 0.999, respectively. The ICCs for BMO-MRW and RNFL thickness in all sectors were >0.991 and >0.998, respectively (Table 3).

In the diseased eyes, the RCs for the average BMO-MRW and RNFL thickness were 9.00 μm (sectoral RCs: 8.43–22.90) and 6.25 μm (sectoral RCs: 5.01–15.67).

For the normal eyes, the RC value for the average BMO-MRW was 4.61 μm and those for the individual sectors ranged from 7.83 to 15.57 μm. The RC value for the average RNFL thickness was 0.92 μm and the values ranged from 1.23 to 2.75 μm for the individual sectors (Table 4).

The CV values for the average BMO-MRW were 0.91% and 0.63% in diseased and normal eyes, respectively. The CV values for the mean RNFL thickness were 1.45% and 0.33%, respectively (Table 4).

## 4. Discussion

The present study investigated the repeatability of BMO-MRW and RNFL thickness measurements in ERM patients with peripapillary involvement compared to healthy controls. The repeatability of BMO-MRW and RNFL thickness measurements, according to the ICC, RC, and CV values, was very good in both diseased and normal eyes.

A few studies reported the repeatability of BMO-MRW measurements in eyes without retinal surface diseases. Enders et al. [13] investigated the intraday consistency of BMO-MRW and RNFL measurements obtained in the morning and afternoon using SD-OCT in glaucoma patients. The mean difference between the intraindividual measurements was 2.95 μm (1.76%) for BMO-MRW and 1.18 μm (1.89%) for RNFL thickness. The ICC for the mean BMO-MRW and RNFL thickness measurements indicated a high degree of consistency between time points for both parameters. Park et al. [14] studied the repeatability of BMO-MRW measurements in non-glaucoma and glaucoma patient groups. For three SD-OCT measurements repeated at the same visit, repeatability of the BMO-MRW measurements was good in both the normal and glaucoma groups based on ICC and CV. In these previous studies, although the repeatability of BMO-MRW was good, comparable to RNFL thickness and normal healthy controls, the studies excluded patients with retinal diseases other than glaucoma.

Measurement repeatability is essential for a test to be considered clinically useful. BMO-MRW measurements can be affected by changes in the retinal surface. ERM with peripapillary involvement can alter the retinal surface around the optic disc and may affect the automatic segmentation of the ILM and inner retina.

Lee et al. [18] reported a high degree of repeatability in CMT, RNFL thickness, and ganglion cell–inner plexiform layer thickness measurements in ERM patients. Even in the group with a high CMT (>450 μm), the CMT and RNFL thickness measurements were highly reproducible, although the ganglion cell–inner plexiform layer (GCIPL) measurements were not. However, this study enrolled patients regardless of peripapillary involvement of ERM; therefore, RNFL thickness, which is a parameter around the optic disc, may not have been affected.

In this study, we used ICC, RC, and CV values as indicators of repeatability. ICCs range from 0 to 1 and can be used to classify the repeatability of a test as follows: <0.5, poor; 0.5–0.75, moderate, 0.75–0.9, good; and >0.9, excellent [19]. The ICCs for the mean BMO-MRW and RNFL thickness in diseased eyes showed that repeatability was excellent in both cases, albeit slightly higher for the BMO-MRW measurements. In addition, the ICCs of BMO-MRW and RNFL thickness in diseased eyes were slightly lower than those of the normal eyes.

The RC values of the mean BMO-MRW and RNFL thickness measurements in diseased eyes were 9.00 and 6.25 μm, respectively. The results indicated that we can expect differences in two repeated BMO-MRW and RNFL thickness measurements of <9.00 and 6.25 μm, respectively, in 95% of subjects. The RC values were lower in normal eyes than those in diseased eyes, also. A small CV value indicates small variability in the difference between two consecutive measurements. Overall, the CV values were lower for the BMO-MRW than RNFL thickness measurements in diseased eyes; the opposite was the case in normal eyes. We compared the CV values of the BMO-MRW and RNFL thickness measurements between the diseased and normal eye of each patient. The mean CV values of average and all sectors of BMO-MRW were not significantly different between the two groups of eyes. However, for the average RNFL thickness, the mean CV value in diseased eyes was significantly lower than that in normal eyes. In addition, the mean CV values of temporal and nasal RNFL thickness were significantly lower in diseased than normal eyes. Thus, the peripapillary membrane may affect the CV value for RNFL thickness measurements to a greater degree in diseased compared to normal eyes, but not for BMO-MRW measurements.

Overall, it seems that repeatability in diseased eyes is slightly inferior to that in normal eyes. CV of average and some sector RNFL thickness was significantly lower in diseased eyes than healthy controls. In addition, in diseased eyes, the RC, CV of average BMO-MRW was 1.9 and 1.4 times greater than that of healthy control eyes, but 6.8 and 4.4 times greater for average RNFL thickness. Therefore, the repeatability of the average RNFL thickness measurements might be more affected by the peripapillary membrane than average BMO-MRW. In other words, the repeatability of BMO-MRW in the ERM patients with peripapillary involvement can be said to be slightly better than that of RNFL thickness.

This study had the limitation that the number of patients was insufficient to perform parametric statistical analysis. Therefore, further studies including larger patient cohorts are needed to confirm our results. Despite this limitation, this study is meaningful because it is the first to investigate the repeatability of BMO-MRW measurements in patients with a retinal disease that can deform the surface of the peripapillary area. In addition, we did not perform manual segmentation of BMO-MRW and RNFL thickness. Comparing auto-segmentation measurements to manual segmentation might help to support the results in the current study. In addition, apart from reproducibility, the BMO-MRW and RNFL thicknesses in peripapillary membrane patients may not be accurate, so it is necessary to pay attention to their interpretation.

In conclusion, BMO-MRW and RNFL thickness measurements showed good repeatability in both ERM patients with peripapillary involvement and normal eyes. Based on the ICC, RC, and CV values, the repeatability of BMO-MRW measurements in these patients was better than that of RNFL thickness. These results need to be taken into account when interpreting BMO-MRW and RNFL thickness in glaucoma patients with peripapillary membrane.

## Figures and Tables

**Table 1:** Baseline characteristics of the patients.

	Diseased Eyes (*n* = 52)	Normal Eyes (*n* = 62)	*p*-Value
Mean age (years)	59.2 (±13.5)	58.6 (±10.0)	0.843 ^†^
Sex			
Male (*n*,%)	30 (57.7%)	41 (66.1%)	0.355 ^‡^
Female (*n*,%)	22 (42.3%)	21 (33.9%)	
Laterality of eye			
Right (*n*,%)	33 (63.5%)	23 (37.1%)	0.005 ^‡^
Left (*n*,%)	19 (36.5%)	39 (62.9%)	
Initial mean BCVA (logMAR)	0.15 (±0.4)	0.28 (±0.32)	0.111 ^†^
Mean spherical equivalent	−0.66 (±2.09)	−0.41 (±1.66)	0.647 ^†^
Mean CMT	333.4 (±88.7) μm	275.6 (±45.2)	<0.001 ^†^

BCVA: best-corrected visual acuity; CMT: central macular thickness; ^†^: independent T-test; ^‡^: Chi-square test.

**Table 2:** The mean BMO-MRW and RNFL thickness measurements in diseased and normal eyes.

		BMO-MRW			RNFL Thickness		
	Sector	Measurement 1	Measurement 2	*p*-Value ^†^	Measurement 1	Measurement 2	*p*-Value ^†^
Diseased Eyes (*n* = 52)	Average	321.6 (±107.0)	320.8 (±104.2)	0.449	124.3 (±42.5)	124.8 (±43.1)	0.729
	TS	327.3 (±105.8)	324.8 (±102.5)	0.206	165.3 (±58.3)	164.4 (±56.9)	0.830
	T	247.0 (±97.1)	245.6 (±96.0)	0.541	106.4 (±45.5)	109.9 (±49.8)	0.272
	TI	355.9 (±104.1)	351.5 (±95.8)	0.174	177.2 (±63.3)	176.6 (±63.8)	0.604
	NI	390.9 (±118.2)	392.0 (±119.1)	0.506	127.1 (±52.7)	129.6 (±54.5)	0.142
	N	329.1 (±126.2)	329.3 (±125.4)	0.814	96.4 (±42.2)	95.8 (±41.3)	0.469
	NS	360.0 (±124.2)	359.6 (±118.4)	0.888	144.6 (±69.7)	145.8 (±61.1)	0.732
Normal Eyes (*n* = 62)	Average	265.9 (±49.4)	266.0 (±49.6)	0.770	101.3 (±13.6)	101.4 (±13.5)	0.240
	TS	266.0 (±43.9)	266.9 (±42.0)	0.482	139.2 (±22.8)	139.4 (±22.7)	0.409
	T	189.9 (±43.7)	189.6 (±44.5)	0.674	76.4 (±10.4)	76.5 (±10.2)	0.201
	TI	299.3 (±69.1)	298.9 (±70.4)	0.810	153.9 (±29.6)	154.2 (±29.5)	0.101
	NI	320.0 (±73.7)	323.4 (±71.9)	0.052	111.1 (±29.6)	111.5 (±29.8)	0.057
	N	280.7 (±60.4)	280.3 (±60.5)	0.515	79.6 (±17.6)	79.6 (±17.7)	0.907
	NS	308.6 (±56.2)	307.6 (±56.0)	0.318	117.4 (±25.5)	117.3 (±25.8)	0.701

RNFL: retinal nerve fiber layer; TS: superotemporal; T: temporal; TI: inferotemporal; NI: inferonasal; N: nasal; NS: superonasal; ^†^: paired t test.

**Table 3:** Intraclass correlation coefficient (ICC) between the two Bruch’s membrane opening-minimum rim width and retinal nerve fiber layer thickness measurements.

	Diseased Eyes				Normal Eyes			
	BMO-MRW		RNFL Thickness		BMO-MRW		RNFL Thickness	
Sector	ICC	95% CI	ICC	95% CI	ICC	95% CI	ICC	95% CI
Average	0.999	0.997–0.999	0.985	0.975–0.992	0.999	0.998–0.999	0.999	0.999–1.000
TS	0.995	0.992–0.997	0.917	0.855–0.952	0.988	0.980–0.993	0.999	0.998–0.999
T	0.993	0.987–0.996	0.939	0.894–0.965	0.994	0.991–0.997	0.998	0.997–0.999
TI	0.986	0.976–0.992	0.995	0.992–0.997	0.993	0.989–0.996	0.999	0.999–1.000
NI	0.998	0.996–0.999	0.986	0.976–0.992	0.991	0.985–0.995	0.999	0.999–1.000
N	0.999	0.999–1.000	0.995	0.991–0.997	0.998	0.997–0.999	0.999	0.998–0.999
NS	0.991	0.984–0.995	0.968	0.945–0.982	0.995	0.992–0.997	0.998	0.997–0.999

BMO-MRW: Bruch membrane opening-minimum rim width; RNFL: retinal nerve fiber layer; ICC: intraclass correlation coefficient; TS: superotemporal; T: temporal; TI: inferotemporal; NI: inferonasal; N: nasal; NS: superonasal.

**Table 4:** Repeatability coefficient (RC) and coefficient of variation (CV) between two the Bruch’s membrane opening-minimum rim width and retinal nerve fiber layer thickness measurements.

	Diseased Eyes				Normal Eyes			
	BMO-MRW		RNFL Thickness		BMO-MRW		RNFL Thickness	
Sector	RC (μm)	CV (%)	RC (μm)	CV (%)	RC (μm)	CV (%)	RC (μm)	CV (%)
Average	9.00	0.91	6.25	1.45	4.61	0.63	0.92	0.33
TS	15.41	1.63	15.67	2.26	12.04	1.58	2.21	0.60
T	14.38	1.79	12.39	3.18	7.83	1.49	1.23	0.59
TI	22.45	2.01	5.01	1.11	11.88	1.51	1.83	0.46
NI	12.47	1.12	7.46	2.28	15.57	2.09	2.21	0.76
N	8.43	0.94	5.24	1.67	7.11	0.95	1.48	0.72
NS	22.90	2.41	11.52	2.78	10.01	1.19	2.75	0.90

BMO-MRW: Bruch membrane opening-minimum rim width; RNFL: retinal nerve fiber layer; RC: repeatability coefficient; CV: coefficient of variation; TS: superotemporal; T: temporal; TI: inferotemporal; NI: inferonasal; N: nasal, NS: superonasal.

## Data Availability

Data is contained within the article.
